# Méningiome en plaque sphéno-orbitaire: à propos d'un cas avec revue de la littérature

**DOI:** 10.11604/pamj.2015.21.159.7190

**Published:** 2015-06-25

**Authors:** Meriem Abdellaoui, Idriss Benatiya Andaloussi, Hicham Tahri

**Affiliations:** 1 Faculté de Médecine et de Pharmacie de Fès, Service d'Ophtalmologie, CHU Hassan II, Fès, Maroc

**Keywords:** Méningiome sphéno-orbitaire, exophtalmie, orbite, spheno-orbital meningiomas, exophtalmia, eye-socket

## Abstract

Le méningiome intra osseux est une variété des méningiomes ectopiques dans lequel les cellules méningothéliales envahissent la paroi osseuse et entraînent une hyperostose. Le méningiome en plaque, variante macroscopique des méningiomes intra osseux, est une tumeur rare et survient fréquemment au niveau de la région sphéno-orbitaire ce qui le confond avec les tumeurs osseuses primitives. Nous rapportons le cas d'une patiente de 50 ans qui présente une exophtalmie avec cécité unilatérale gauche d'installation progressive depuis un an. L'examen trouve une exophtalmie axile, indolore et non réductible ainsi qu'une limitation de la motilité oculaire dans tous les sens du regard. La palpation montre une masse temporale gauche dure et adhérente à l'os. L'examen du fond d’œil trouve un œdème papillaire gauche. Le scanner montre une lésion ostéocondensante temporo-sphéno-orbitaire gauche avec envahissement locorégional. Le diagnostic préopératoire fut une tumeur osseuse essentiellement maligne primitive ou secondaire. L’étude histologique a révélée un méningiome meningothélial de type en plaque. La patiente a bénéficié d'une exérèse avec reconstruction chirurgicale. Aucune récidive n'a été notée après 1 an de recul.

## Introduction

Le méningiome intra osseux est une variété des méningiomes ectopiques dans lequel les cellules méningothéliales envahissent la paroi osseuse et entraînent une hyperostose [1]. Le méningiome en plaque, variante macroscopique des méningiomes intra osseux, survient fréquemment au niveau de la région sphéno-orbitaire [1]. Il naît au niveau des méninges le plus souvent juxta-sphénoïdales, et envahit le contenu orbitaire soit par la fissure orbitaire supérieure ou le canal optique, soit plus fréquemment après envahissement osseux [1, 2]. Le méningiome sphéno-orbitaire est très rare [2, 3] et il est souvent confondu en préopératoire avec les tumeurs osseuses primitives [2]. Nous rapportons un cas rare de méningiome en plaque sphéno-orbitaire colligé au service d'ophtalmologie du CHU HASSAN II de Fès, Maroc.

## Patient et observation

**Figure 1:**
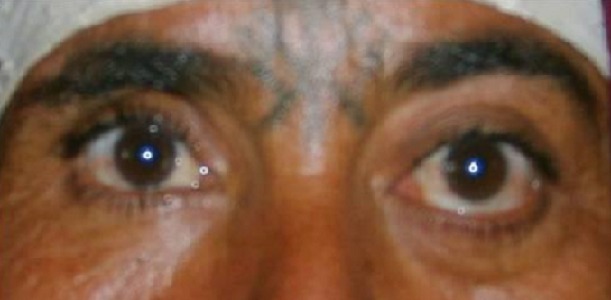
Photo de face montrant l'exophtalmie gauche

**Figure 2:**
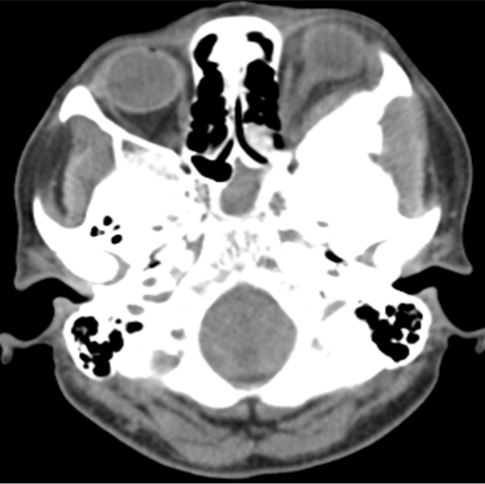
Coupe scannographique axiale montrant la tumeur osseuse sphéno-orbitaire

**Figure 3:**
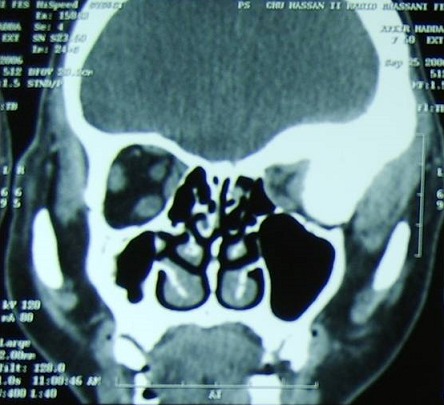
Coupe scannographique coronale montrant l'envahissement intra-orbitaire et des tissus mous de la tumeur osseuse

**Figure 4:**
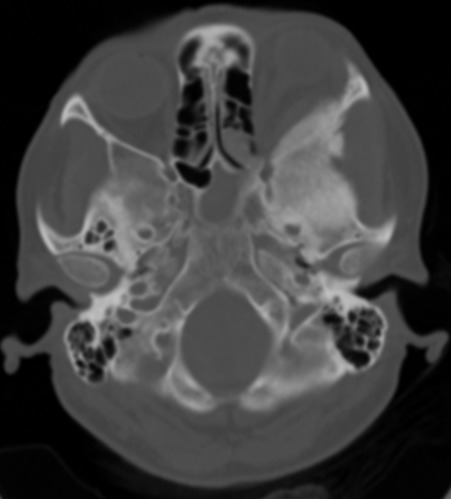
Coupe scannographique axiale en fenêtre osseuse montrant l'hyperostose corticale de la tumeur sphéno-orbitaire

**Figure 5:**
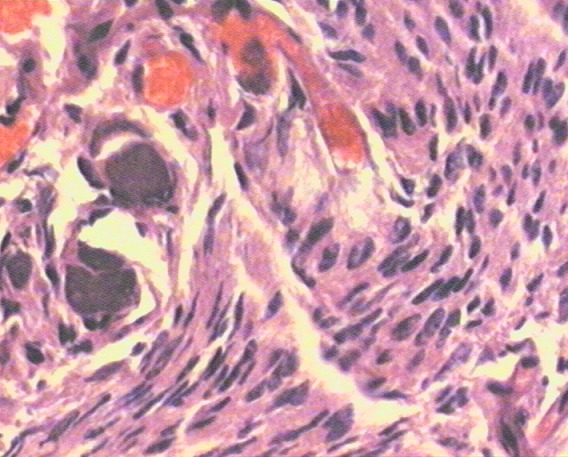
Aspect histologique de la tumeur osseuse en faveur du méningiome en plaque

Mme H.A âgée de 50 ans consulte en ophtalmologie pour une exophtalmie avec cécité unilatérale gauche associées une tuméfaction de la région temporale du même côté, évoluant progressivement depuis une année sans notion de traumatisme crânien. L'examen trouve une exophtalmie axile, indolore, non réductible et non pulsatile (Figure 1) ainsi qu'une limitation de la motilité oculaire dans tous les sens du regard. La palpation montre une masse temporale gauche dure et adhérente à l'os. L’évaluation de l'acuité visuelle révèle une absence de perception lumineuse et l'examen du fond d’œil trouve un œdème papillaire. L'examen de l’œil droit est normal. La patiente ne présente aucun déficit neurologique. Le scanner montre une lésion ostéocondensante temporo-sphéno-orbitaire gauche avec envahissement endocrânien, orbitaire et des parties molles temporales (Figure 2, Figure 3, Figure 4). Le diagnostic préopératoire fut une tumeur osseuse essentiellement maligne primitive ou secondaire, cependant une dysplasie fibreuse reste également un diagnostic probable. La biopsie osseuse a révélée après étude immunohistochimique, un méningiome méningothelial de type en plaque (Figure 5). La patiente a bénéficié d'une exérèse chirurgicale avec reconstruction durale et pariétale en neurochirurgie. Aprés un recul ophtalmologique post-chirurgical d'une année aucune récidive n'a été notée, cependant une atrophie optique s'est rapidement installée.

## Discussion

Le terme de méningiomes ectopiques regroupe l'ensemble des méningiomes qui se développent en dehors des méninges, ils sont appelés également méningiomes extracranien, extraneuraxials, cutanés ou intra osseux [1]. Pour éviter toute confusion Lang et al ont proposé de les nommer communément les méningiomes extraduraux primitifs [4]. Ce type de méningiomes est rare [1, 2, 4], l'incidence rapportée est de 1% à 2% de l'ensemble de ces tumeurs [4, 5]. Soixante huit pourcent des méningiomes extraduraux primitifs touchent la région céphalique [5]. Les régions sphéno-orbitaire et frontopariétale sont les localisations les plus fréquentes des méningiomes intra osseux [1, 4, 5]. Il existe différentes hypothèses concernant l'origine de ces méningiomes intraosseux céphaliques [1, 5]; le développement de cette tumeur se ferait à partir de méningocytes ectopiques ou bien de cellules des capillaires arachnoïdiennes qui seraient piégées dans les sutures crâniennes durant le développement embryonnaire. La seconde hypothèse serait également un piégeage des cellules méningothéliales dans les sutures ou bien dans les traits de fractures crâniens secondaires à un traumatisme, cependant ce dernier était absent chez notre patiente. Crawford et al [6] trouve seulement 5 parmi 36 cas avec méningiome intra osseux primitif qui ont une histoire de traumatisme crânien au niveau de la région ou se localise la tumeur. Le méningiome intra osseux touche aussi bien les hommes que les femmes sans prédominance de sexe, il survient en général au cours de la dernière décade de la vie [1, 4]. Cliniquement, les patients avec méningiome sphéno-orbitaire se présentent typiquement avec une exophtalmie progressive et indolore ainsi q'une baisse de l'acuité visuelle très lente pouvant aboutir à la cécité sans manifestations neurologiques associées [1, 2, 4] ceci concorde avec les données cliniques de notre patiente.

Histologiquement le méningiome en plaque sphéno-orbitaire est une tumeur bénigne dont la croissance est très lente [1]. Le terme en plaque désigne qu'il s'agit d'une variante macroscopique des méningiomes, cette appellation est utilisée pour la première fois par Cushing [1, 7] faisant référence à l'aspect en plaque ou en cartiers de la tumeur. La radiographie standard est très limitée dans le diagnostic des méningiomes intra osseux sphéno-orbitaires à cause de la superposition des parois osseuses. La tomodensitométrie avec fenêtre osseuse est nécessaire pour détecter l'hyperostose corticale de la tumeur ainsi que l'extension intra et extra osseuse. L'hyperostose est l'image radiologique la plus fréquente (59%) mais l'ostéolyse est également rapportée dans 35% de même qu'une image mixte dans 6% des cas [1, 4, 6]. L'IRM avec injection de gadolinium incluant la séquence T1 avec suppression de la graisse parait plus performante que le scanner dans la suggestion du méningiome en plaque sphéno-orbitaire [7–9], en plus elle permet de bien délimiter l'extension tumorale surtout au niveau des tissus mous et du parenchyme cérébral. Le diagnostic différentiel du méningiome en plaque sphéno-orbitaire inclus essentiellement le cancer métastatique, l'ostéosarcome et la dysplasie fibreuse de l'os [1, 2, 6, 7, 10]. Le méningiome en plaque sphéno-orbitaire est une tumeur bénigne qui, bien que d´évolution lente, impose une intervention chirurgicale à partir du moment où le diagnostic est posé [3, 9]. La craniectomie fronto-temporo-sphénoïdale avec résection de tous les tissus envahis à savoir la dure mère, le muscle et les tissus intra orbitaires, avec reconstruction durale et pariétale est le traitement de choix [11–13]. Une radiothérapie adjuvante est recommandée chez les patients qui présentent des lésions résiduelles symptomatiques et/ou augmentant de volume [3, 11, 12]. Les chances de guérison étant inversement proportionnelles au degré d´extension de la tumeur. La lenteur d´évolution facilite la surveillance des reliquats tumoraux et/ou le dépistage des récidives par la réalisation d´un contrôle IRM annuel. Lorsqu´il s´y associe une atteinte de la fonction visuelle, l´acuité visuelle préopératoire ainsi que la durée d´évolution constituent des éléments à valeur pronostique quant à la récupération fonctionnelle post-opératoire [3, 9].

## Conclusion

Les méningiomes temporo-sphéno-orbitaires en plaques sont des tumeurs rares, d’évolution lente, responsables d'une symptomatologie essentiellement ophtalmologique. L'atteinte de la fonction visuelle est un facteur de mauvais pronostic. Ainsi Il convient de bien informer le patient que l'intervention chirurgicale ne garantit pas toujours la récupération visuelle.
